# Double-blind acupuncture needles: a multi-needle, multi-session randomized feasibility study

**DOI:** 10.1186/s40814-018-0265-9

**Published:** 2018-04-13

**Authors:** Judith M. Schlaeger, Nobuari Takakura, Hiroyoshi Yajima, Miho Takayama, Alana D. Steffen, Elizabeth M. Gabzdyl, Robyn A. Nisi, Kathleen McGowan Gruber, Jason M. Bussell, Diana J. Wilkie

**Affiliations:** 1 Department of Women, Children and Family Health Science, College of Nursing, University of Illinois at Chicago, 845 S. Damen (M/C 802), Chicago, IL 60612 USA; 2 Department of Acupuncture and Moxibustion, Faculty of Health Sciences, Tokyo Ariake University of Medical and Health Sciences, 2-9-1 Ariake, Koto-ku, Tokyo, 135-0063 Japan; 3 Bloomberg School of Public Health, Johns Hopkins University, 615 N. Wolfe Street, Baltimore, MD 21205 USA; 4 Department of Biobehavioral Nursing Science, College of Nursing, University of Florida, 1225 Center Drive, Gainesville, FL 32610 USA

**Keywords:** Acupuncture, Double-blind acupuncture needles, Vulvodynia, Placebo, Provoked vestibulodynia, Vulvar vestibulitis, Pain

## Abstract

**Background:**

Efficacy of acupuncture is difficult to demonstrate without a feasible double-blind milieu. Double-blind acupuncture needles have been validated in single session protocols with one or two needles but not been tested in a protocol requiring many needles and repeated sessions.

**Methods:**

We determined the feasibility of a 13-needle, 10-session study protocol. Feasibility focused on (1) enrolling and retaining participants; (2) two acupuncturists accurately implementing a double-blind, multi-needle, multi-session protocol; (3) participants completing measures; and (4) protocol acceptability to participants. In this double-blind randomized controlled pilot study, participants were randomized 1:1 to a penetrating needle group or a skin-touch placebo control group.

**Results:**

Six women with vulvodynia (mean age 31.5 ± 8 years; five white, non-Latina, one black/African American) met the eligibility requirements, consented to participation, and were enrolled. All six participants (100%) completed the 10-session study protocol in 5 weeks without missing any treatment sessions. Per observed checklist documented technique, two acupuncturists flawlessly administered the 13-needle, 10-session acupuncture protocol; no needles malfunctioned. Six participants attended all sessions and completed 99% of measurement items. One participant did not like acupuncture (60% acceptability score) and five liked acupuncture (100% acceptability scores); the mean acceptability score was 93.3%.

**Conclusion:**

Study feasibility was supported. This protocol can be used in a double-blind efficacy trial of acupuncture for vulvodynia.

**Trial registration:**

ClinicalTrials.gov, NCT02704234. Registered 30 November 2015.

**Electronic supplementary material:**

The online version of this article (10.1186/s40814-018-0265-9) contains supplementary material, which is available to authorized users.

## Background

Efficacy of acupuncture is difficult to demonstrate without a feasible double-blind milieu, but double-blind acupuncture needles have been validated only in single session protocols with one or two needles. It is unknown whether feasibility of a protocol requiring many needles and repeated sessions is possible or what procedures and steps are needed to implement a multi-needle, multi-session, double-blind acupuncture needle study protocol. Such pilot study testing is an essential prerequisite to rigorous implementation in a randomized controlled trial (RCT), so problems may manifest and be rectified prior to initiating a large RCT. The purpose of this feasibility study was to test such a protocol before it is implemented in a future RCT efficacy trial for the treatment of vulvodynia.

Although non-penetrating blunt tip placebo/sham needles [1, 2] or superficial insertion to non-acupuncture points were employed in acupuncture clinical trials, those needles blind only the participant, namely, they are single-blind needles. Single-blind sham acupuncture may have up to a 38% placebo effect as unblinded practitioner bias may influence treatment outcomes [3].

To explore whether or not acupuncture can be efficacious in clinical trials, the best research design is one that uses double-blind acupuncture needles, those that are placed at the acupuncture site but penetration is unknown to either the therapist or the patient [4, 5]. Desirably, the non-penetrating control needles must be inert or as close to inert as possible and ensure double-blindness. Therefore, to the acupuncturist and participant, they must (1) look identical to real penetrating needles, (2) be inserted in the same manner as real needles for the experimental condition being treated, and (3) feel identical to real penetrating needles upon insertion [5].

We identified a total of eight studies [4, 6–12] focused on the validity of the double-blind needles. In seven studies of acupuncturists and participants remaining blind, results indicated that acupuncturists and participants were unable to discern better than chance the penetrating needles versus non-penetrating skin-touch placebo needles. In an eighth and only study, researchers identified that larger percentages of acupuncturists and participants than chance correctly guessed the type of needle that was inserted [12]. In this study, the double-blind acupuncture needles were used for a one session treatment of post-operative pain status post third molar extraction with participants receiving either all penetrating or all skin-touch placebo needles at five different acupuncture points. Needle type was correctly identified by 61% of participants and 83% of acupuncturists. Factors that may have contributed to a higher correct identification rate included that needles were, in the Chinese style, rotated vigorously, three separate times by acupuncturists who were trained and practice Chinese style acupuncture, which has a much more aggressive needle insertion technique. Indeed, the double-blind needles were developed for a gentler Japanese acupuncture style insertion technique. In Japanese acupuncture, needles are seldom aggressively manipulated using one-way rotation during needle retention. Therefore, for the world’s only double-blind needles, researchers have demonstrated that a double-blind milieu can be maintained in single session application of one or more pairs of double-blind needles (penetrating and skin-touch placebo) [4, 6–10]. However, there is a paucity of evidence for using these double-blind needles in a multi-needle, multi-session research study.

Therefore, the purpose of this multiple needle, multi-session pilot study of acupuncture for the treatment of vulvodynia was to determine the protocol feasibility. Specifically, the specific aims focused on the feasibility of (1) enrolling and retaining at least 80% of the participants, all of whom completed all study measures and reported the 10-session study protocol as acceptable (scores ranging > 10 (80%), indicating high acceptability). We also aimed to implement an observed fidelity checklist, describe technical issues of two acupuncturists using the protocol, and identify challenges to implementing the protocol. In summary, this study focused on recruitment, retention, data collection, and intervention procedural problems. Using these findings in the future, we will advance this work to a double-blind RCT study focused on efficacy of the protocol for the pain-related symptoms of vulvodynia.

## Methods

### Study design and setting

**Fig. 1:**
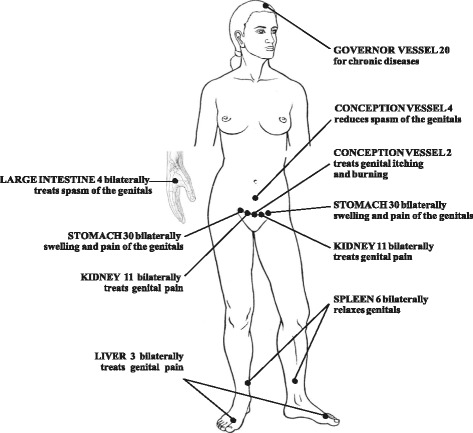
Standardized acupuncture protocol for the treatment of vulvodynia [13]; figure reprinted with permission from *The Journal of Chinese Medicine*

This feasibility study mimicked the design of the planned RCT with double-blind placebo control. We used a 13-needle, 10-session, twice-weekly, standardized acupuncture treatment protocol (Fig. 1) that demonstrated efficacy in a prior wait-list controlled trial [13]. This feasibility study was conducted at an acupuncture clinic in suburban Chicago. The principal investigator and one other acupuncturist, both licensed in Illinois, administered the treatments. The University of Illinois at Chicago’s Institutional Review Board approved this study. Our study design, intervention, and reporting adhered to the Consolidated Standards of Reporting Trials (CONSORT) 2010 Checklist of Information to Include when Reporting a Pilot or Feasibility trial (Additional file 1).

### Participants and randomization

Participants were recruited between December 2015 and March 2016 from advertisements (on the National Vulvodynia Association (NVA) website, the Chicago Reader newspaper) and posted flyers. A recruitment letter was sent to three urogynecologists, eight obstetrician-gynecologists, and certified nurse midwives who had all referred other women with vulvodynia to our previous vulvodynia study. Prospective participants self-referred to the study by telephoning or emailing the principal investigator or research assistant.

Inclusion criteria based on self-report were English speaking, 18 years old up to menopause, previously diagnosed with generalized or localized vulvodynia by their obstetrician-gynecologist, urogynecologist, or minimally invasive gynecologic surgeon. Participants were allowed to continue all medications for vulvodynia and other medical conditions as previously prescribed. Exclusion criteria included pregnancy, menopause, interstitial cystitis, irritable bowel syndrome, untreated vaginitis, cervicitis, pelvic inflammatory disease, any other pelvic pathology causing pain, and concomitant physical therapy, biofeedback, massage, or additional acupuncture. Participants presented proof of their diagnosis from their healthcare provider upon admission to the study. No medical records were reviewed nor physical/gynecologic exams performed to confirm inclusion/exclusion criteria.

Participants were randomized 1:1 to a penetrating needle group or a skin-touch placebo control group. The statistician used a random number function in a spreadsheet program and randomly sorted study IDs into the two treatment groups. Those ID numbers were affixed to the appropriate pack of double-blind acupuncture needles (three penetrating and three non-penetrating touch placebo needles). Participants were assigned sequentially to the IDs according to the order they were enrolled in the study. Neither the research assistant, participants, nor acupuncturists were informed of participant group allocation until all six participants completed the study.

### Intervention protocol

**Fig. 2:**
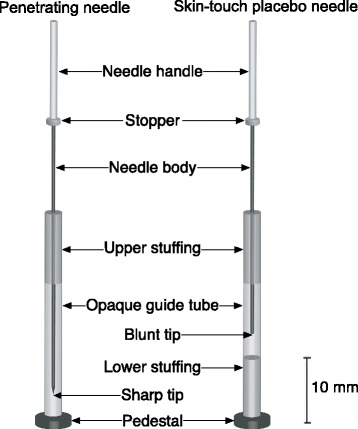
Mechanisms of double-blind acupuncture needles: penetrating and skin-touch placebo

**Fig. 3:**
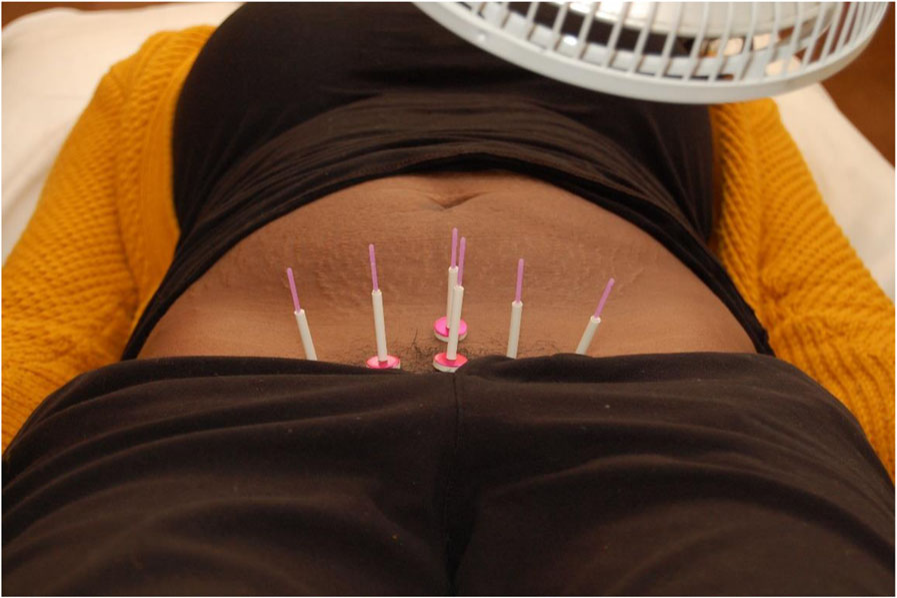
Inserted double-blind needles with inner guide tubes and pedestals

Together in Tokyo over a 40-h period, the inventor of the double-blind acupuncture needle and the principal investigator developed this study protocol. They produced three video demonstrations, which the inventor recorded as the principal investigator performed each step of the protocol. With the double-blind needles, the needle handle is preset to be held in place by the top of the guide tube with the needle suspended within. The guide tube is placed over the acupuncture point flush with the skin. The index finger of the needling hand taps the needle handle, which thrusts the needle downward through the guide tube in order to penetrate the skin. The non-penetrating skin-touch blunt tip placebo needle travels through stuffing within the lumen of the inner guide tube (Fig. 2). This skin-touch placebo needle slightly extends beyond the bottom of the inner guide tube to touch the skin but does not penetrate. This process produces the feeling of skin being punctured for the participant and the needle moving through tissue for the acupuncturist; thus; a double-blind milieu is created and maintained. The outer guide tube for both types of double-blind needles is then removed; an opaque inner guide tube remains to maintain the blind. Both types of needles are then inserted further using an alternating twirling technique. The bottom of the inner guide tube inserts into a lamellar paper pedestal with adhesive on its underside and enables the whole assembly to stick to the skin, remain upright, and maintain the blind (Fig. 3).

A trained research assistant was present at every acupuncture session and used the protocol checklist to document the acupuncturists’ adherence to the protocol (fidelity of the intervention delivery). Both acupuncturists each have been practicing for 14 years. The acupuncturist and participant were reminded not to speak to one another except as needed to deliver the intervention safely. Although the Japanese style is less prone to unblinding, we employed the Chinese style of acupuncture to be consistent with our previous pilot study [13]. In the principal investigator's previous unblinded wait list controlled RCT for the treatment of vulvodynia [13], needles were retained for 30 min and aggressively rotated in a counter-clockwise direction, three separate times during retention. We were concerned that for this feasibility study, aggressively rotating the needles three separate times would cause bleeding and/or bruising and thus unblind the participant and/or acupuncturist. Therefore, for the first four participants, the first acupuncturist (principal investigator) gently rotated all needles in a counter-clockwise direction at 15 and 30 min, and just before needle removal at 45 min after insertion. This twisting-rotating (one-way rotation) technique is commonly used in Chinese style acupuncture to induce the needling (*de qi*) sensation which is considered to be therapeutic and to stimulate the transmission of the energy (*qi*) through the acupuncture channels [14, 15]. We increased the needle retention time to 45 min to compensate for gentler needle rotation (thus less stimulation of the *qi* and transmission through the acupuncture channel), so a therapeutic effect could still be achieved. The research assistant remained with the participant for the whole treatment session. The first acupuncturist left the room after the needles were initially inserted, but returned just to rotate the needles and then left again. The first acupuncturist administered acupuncture to the first four participants according to the protocol.

Challenges to the implementation of the study protocol were identified (see the “Results” section for specifics), and it was then modified after completion of the fourth participant’s 10th acupuncture treatment. Specifically, the three needle rotations at 15, 30, and 45 min after needle insertion were discontinued to minimize bleeding and/or bruising to protect blinding, and the requirement that the research assistant remain in the room was discontinued due to the participants’ talking intermittently during and after the acupuncture treatments. The first acupuncturist taught the modified protocol to the second acupuncturist who administered acupuncture to two study participants. The first acupuncturist observed the second administer all acupuncture treatments to ensure study fidelity using the protocol checklist.

### Procedures

The research assistant described the study and obtained signed informed consent from participants. Participants were informed that they would receive either all penetrating acupuncture needles or all non-penetrating skin-touch placebo needles for the whole 13-needle, 10-session acupuncture protocol to determine if they could tell which needles they received over the course of the study. Risks and benefits, the voluntary nature of participation, and the capacity to discontinue participation without consequences at any time were discussed.

Participants received acupuncture two times per week for 5 weeks for a total of 10 treatments. To enhance retention, we made reminder calls to all participants the night before their scheduled sessions and provided acupuncture to participants based on their availability (during day, evening, Saturday, and Sunday hours). If participants received placebo acupuncture, they were offered 10 free regular acupuncture treatments after all subjects completed the study.

### Measures

For the feasibility indicator—missing data, the following valid and reliable measures were self-administered on an Internet-based tablet: (1) PAIN*Report*It® [16–18], (2) Female Sexual Function Index [19], (3) Short Form-36 Health Survey [20], (4) Pittsburgh Sleep Quality Index [21], and (5) Protocol Acceptability Scale for Treating Vulvodynia with Acupuncture [16, 18]. We instructed women to allot one and one-half hours to complete all questionnaires and receive acupuncture for the first and tenth (last) sessions and 1 h for the second through ninth sessions. Additional details are provided for the measure from which results are reported in this article.

The Protocol Acceptability Scale for Treating Vulvodynia with Acupuncture (Acceptability) [16, 18] assessed the acceptability of the study intervention and all the measures being completed on Internet-based tablets. The Acceptability scale is a 10-item self-report measure about the study acceptability to the participants. Response options include the following: too hard, somewhat hard, or not hard to participate in this study; too hard, somewhat hard, or easy to understand the study protocol; too hard, somewhat hard, or easy to see the study questions; too rushed, somewhat rushed, or not rushed to complete this study; enjoyed, somewhat enjoyed, or did not enjoy being in this study; I liked, I liked or did not like, or I did not like receiving acupuncture; acupuncture was painful or not painful; I would or would not get acupuncture again; others will, somewhat, or not enjoy being in this study; and the study was too long, too short, or just the right length. This scale has been used previously to determine the acceptability of a different intervention protocol. It is reliable and valid (mean acceptability score was 11 ± 2 for a 13-item computer acceptability score) and stable over 4 weeks showing test-retest reliability [16, 18]. The possible total score ranges from zero to 20, and higher scores indicate greater acceptability of the study. A priori, the 10-session study protocol was deemed to have sufficient acceptability to the study participants if the total mean acceptability scale score was > 80% of the maximum score.

## Analysis

Descriptive statistics were used to analyze the data. Frequencies and percentages were calculated to determine the feasibility of enrolling and retaining at least 80% of the participants, all of whom completed all study measures and reported the 10-session study protocol as acceptable (scores ranging > 16 [80%], indicating high acceptability). We also aimed to implement an observed fidelity checklist, describe technical issues of two acupuncturists using the protocol, and identify challenges to implementing the protocol.

## Results

### Recruitment, enrollment, and retention

**Fig. 4:**
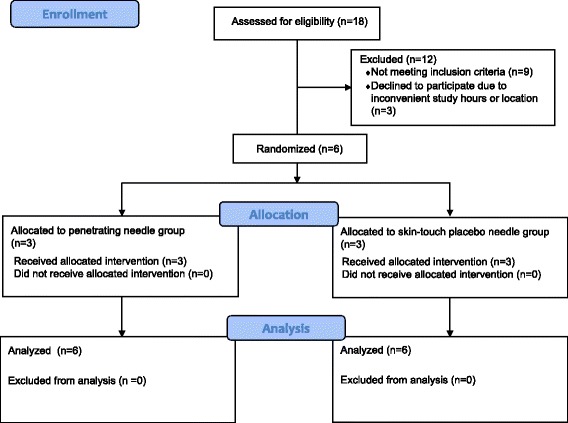
Consort flow diagram

Eighteen women were referred and screened for eligibility (Fig. 4): 14 from the National Vulvodynia Association website, two from medical providers, and two from posted study recruitment flyers. Nine women were ineligible due to the presence of concomitant diagnoses of menopause (six), interstitial cystitis (two), and endometriosis (one); three women were eligible but declined to participate due to either inconvenient study hours or location.

Six women with vulvodynia (mean age 31.5 ± 7.99 years; five white, non-Latina; one black/African American) met the eligibility requirements, consented to participation, and were enrolled. Of the six women who were enrolled, five were acupuncture naive. Three participants were randomly assigned to each of the two groups. All six participants (100%) completed the 10-session study protocol in 5 weeks without missing any treatment sessions.

### Missing data

The participants completed all questionnaires. The missing data rate was less than 1%. Two of the 30 (0.7%) weekly completed Female Sexual Function Index questionnaires were not filled out due to research assistant error. All the other study questionnaires were completed in their entirety.

### Protocol acceptability

Averaged across the six participants, the acceptability score was 93.3%. Five participants reported 100% acceptability indicating that they believed participating in the study was not hard at all, enjoyed being in the study, liked acupuncture, thought it was not painful, would get it again, and answered that others would enjoy being in the study. One participant reported 60% acceptability, and she found participating in the study was somewhat hard, did not enjoy being in the study, did not like acupuncture, thought it was painful, would not get it again, and answered that others will somewhat enjoy being in the study.

### Intervention fidelity

All participants received acupuncture within the allotted time for the first and last session (one and one-half hours) and the second through ninth sessions (1 h). There were 60 completed acupuncture sessions with 13 needles per session. All 130 needles per participant (a total of 780 needles) were inserted, retained, remained in place (even the placebos), and removed according to the study protocol; no needles malfunctioned. The trained research assistant was present at every acupuncture session and used the protocol checklist to document the acupuncturists’ 100% adherence to the initial protocol used by the first acupuncturist and the second modified protocol checklist used by the second acupuncturist (fidelity of the intervention delivery).

### Challenges to protocol implementation

We identified challenges to implementing the double-blind acupuncture treatment protocol and potential threats to maintaining the blind. The first challenge was that two of the first four participants who were needled each had one small bruise after one session, and after unblinding, it was noted that they both were allocated to the penetrating needle group. Bruising in the first participant caused her to become unblind but not the first acupuncturist; bruising in the third participant did not cause the participant to become unblind but caused the first acupuncturist to become unblind. After the second acupuncturist was trained and followed the modified protocol, the last two participants did not have needles rotated after the initial insertion and they did not have bruising.

A second challenge was that three of the first four participants talked intermittently during their treatment sessions to the research assistant who remained in the room for the entire treatment session. Two of the four participants specifically commented about how much better they felt due to receiving acupuncture treatments. The protocol was then modified, and the second acupuncturist and the observer left the room after the needles were inserted and returned 45 min later, one of the two participants frequently talked upon their return. Neither acupuncturist admitted that participant talking caused them to become unblind; however, unnecessary talking is a threat to study validity.

## Discussion

We successfully recruited, enrolled, and retained all study participants, well above our goal of 80%. Missing data was minimal, and the burden of study participation was on average acceptable and well above the 80% criterion, with only one subject below that threshold. Intervention fidelity was high, but we identified challenges that required slight modification of the protocol to support the double-blind condition for this standardized, 13-needle, 10-session acupuncture treatment protocol. After the changes, the challenges did not manifest again.

It is not common in multi-session intervention studies to have 100% retention. We attributed our 0% attrition rate to the fact that all six women with vulvodynia in our study had felt they had tried all available Western medical treatment options and still had vulvar pain and dyspareunia. This is our second study with no attrition as we had no attrition in our 36 participant wait-list control study as well [13]. For both studies, our retention strategies (reminder calls the night before their scheduled sessions and sessions conducted during day, evening, Saturday, and Sunday hours) were highly successful. The dire need of the women with vulvodynia may also have contributed to the high retention rate. There are no consistently effective treatment options for women with vulvodynia [22], and only 22–26% will attain remission [23]. The women expressed interest in advancing vulvodynia research with hopes of better therapies. Five participants stated they were not only hoping to obtain pain relief but also were motivated to participate in advancing vulvodynia research. The one participant that did not enjoy being in the study, did not like acupuncture, thought it was painful, and would not get it again stated she did not discontinue the study because she felt strongly about the need to help advance vulvodynia research so “women with vulvodynia did not have to suffer.”

Finally, our low attrition rate may have been related to free acupuncture treatments offered to the control group. In the USA, the cost of acupuncture, which is usually not covered by most health insurance policies, ranges from $65 to $120 per treatment [24]. This pilot study enabled women in the control group to receive 10 free acupuncture treatments on completion of the study.

This feasibility study allowed us to identify the problem and the cause of bruising, needle rotation, which is a common method used in Chinese style acupuncture. It has the potential to unblind both participant and acupuncturist. The modified protocol rectified the bruising problem and eliminated further challenges to its implementation. However, eliminating needle rotation as part of the protocol may reduce the efficacy of the treatment. We made the decision in favor of blinding because we believed that the reduction in efficacy would be negligible and that the scientific rigor of maintaining the blind is more important. Additional research is needed to verify that the protocol minimizes the potential for bruising to unblind the acupuncturist. Our sample size of six was too small to determine if in future studies, only the participants allocated to the penetrating needle group will bruise. Also, it cannot be inferred that participants who bruise become unblind; only one of ours did. She was acupuncture naive and did not know what to expect.

We identified that extraneous participant talking had potential to unblind both participant and acupuncturist. We also modified the protocol to reduce participants’ verbal contact with study personnel. This impromptu speaking may have been due to a tendency of American patients to value an interpersonal relationship with their healthcare provider [25] or anxiety as five out of six participants were acupuncture naive upon admission to the study.

The fidelity of the intervention delivery was easily monitored with the protocol checklist. None of the double-blind needles inserted malfunctioned. The modified, standardized protocol removed social support, which could itself be an intervention, and communication about treatment effects, which could unblind the acupuncturist. This protocol therefore has the potential to test the effects of the needling procedure in a rigorous way.

There was an acceptable amount of missing data. All tablet-based measures and acupuncture treatments were completed within the allotted time per session.

A limitation of our study is its small sample size. We attribute our small sample size to the large expense of conducting this type of research and the intramural funding to support it. Nonetheless, we were able to insert a total of 780 needles and administer 60 treatments, which will provide important insights for implementing a larger double-blind RCT focused on efficacy.

Because the goal of this study was to determine the feasibility of the procedure, inclusion criteria based on self-report and confirmation of diagnosis from a healthcare provider were sufficient for enrollment into this study. Also, minimum pain intensity scores were not necessary for enrollment. For a future efficacy trial, potential participants should have physical/gynecologic exams performed as well as pain intensity scores measured to determine if minimum pain thresholds have been met (to enable measurement of a clinical reduction in pain at post-test) as part of study inclusion/exclusion criteria.

## Conclusion

This feasibility trial was necessary since it was the first-time double-blind acupuncture needles were used in a multi-needle, multi-session study. We demonstrated the feasibility of using these highly innovative double-blind needles in a double-blind RCT of our 13-point, 10-session acupuncture protocol for the treatment of vulvodynia. Such a study will allow determination of acupuncture effects from placebo effects with potential to offer a new treatment for the millions of women suffering from the chronic pain-related symptoms of vulvodynia. These findings will also advance acupuncture treatment research regarding the use of a double-blind research design.

## Additional file

Additional file 1: Consolidated Standards of Reporting Trials (CONSORT) 2010 Checklist of Information to Include when Reporting a Pilot or Feasibility trial. (DOC 226 kb)
